# Cerebrovascular and Neurological Dysfunction under the Threat of COVID-19: Is There a Comorbid Role for Smoking and Vaping?

**DOI:** 10.3390/ijms21113916

**Published:** 2020-05-30

**Authors:** Sabrina Rahman Archie, Luca Cucullo

**Affiliations:** 1 Department of Pharmaceutical Sciences, Texas Tech University Health Sciences Center, Amarillo, TX 79106, USA; Sabrina.Archie@ttuhsc.edu; 2 Center for Blood-Brain Barrier Research, Texas Tech University Health Sciences Center, Amarillo, TX 79106, USA

**Keywords:** SARS-CoV-2, COVID-19, cerebrovascular, neurological, smoking, CNS, blood-brain barrier

## Abstract

The recently discovered novel coronavirus, SARS-CoV-2 (COVID-19 virus), has brought the whole world to standstill with critical challenges, affecting both health and economic sectors worldwide. Although initially, this pandemic was associated with causing severe pulmonary and respiratory disorders, recent case studies reported the association of cerebrovascular-neurological dysfunction in COVID-19 patients, which is also life-threatening. Several SARS-CoV-2 positive case studies have been reported where there are mild or no symptoms of this virus. However, a selection of patients are suffering from large artery ischemic strokes. Although the pathophysiology of the SARS-CoV-2 virus affecting the cerebrovascular system has not been elucidated yet, researchers have identified several pathogenic mechanisms, including a role for the ACE2 receptor. Therefore, it is extremely crucial to identify the risk factors related to the progression and adverse outcome of cerebrovascular-neurological dysfunction in COVID-19 patients. Since many articles have reported the effect of smoking (tobacco and cannabis) and vaping in cerebrovascular and neurological systems, and considering that smokers are more prone to viral and bacterial infection compared to non-smokers, it is high time to explore the probable correlation of smoking in COVID-19 patients. Herein, we have reviewed the possible role of smoking and vaping on cerebrovascular and neurological dysfunction in COVID-19 patients, along with potential pathogenic mechanisms associated with it.

## 1. Introduction

Coronavirus disease 2019 or COVID-19 is an infectious disease caused by a recently discovered form of coronavirus known as severe acute respiratory syndrome coronavirus-2 (SARS-Cov-2) [1,2]. The outbreak of this virus first appeared in Wuhan city of Hubei province in China in December 2019 [3]. On March 11, 2020, it was declared as a pandemic by the World Health Organization (WHO) [4]. As of May 28th 2020, a total number of 5,792,874 coronavirus cases, including 357,479 deaths, have been reported all over the world [5]. This virus is spreading among all populations so rapidly that, in just three months, the USA has become the epicenter, having 1,745,803 confirmed cases, including 102,107 deaths. The number of confirmed cases seems to indicate a steady increment over time and these numbers can be forecasted using several mathematical models available for COVID-19 [5,6,7,8].

**Figure 1:**
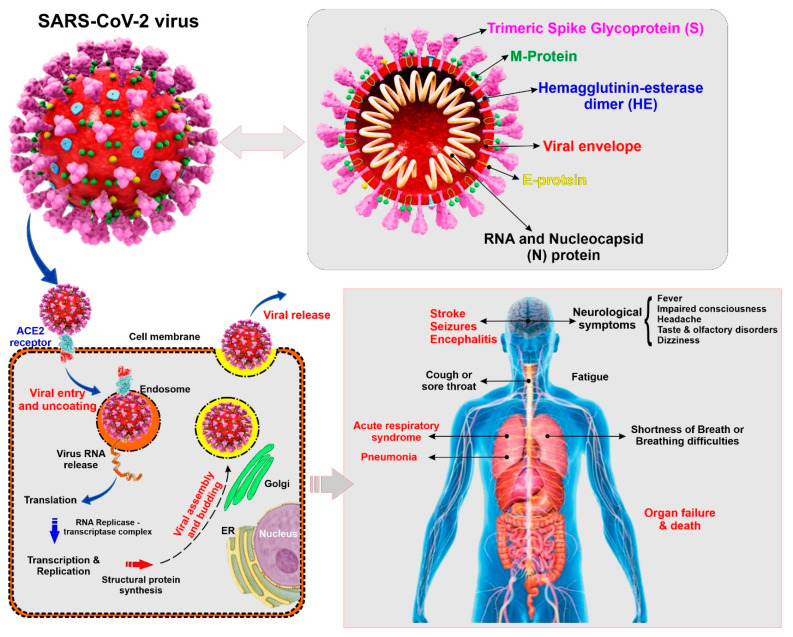
Illustrative view of the SARC-CoV-2 virus structural components and known modality of viral entry into the cells. The scheme also provides a summary panel of the potential health impact on the human body specific to lung and the CNS. (ACE2: Angiotensin converting enzyme 2, ER: Endoplasmic reticulum)

Coronaviruses belong to the subfamily Coronavirinae (family Coronaviridae; order Nidovirales), containing four genera named, *Alphacoronavirus*, *Betacoronavirus*, *Gammacoronavirus*, and *Deltacoronavirus* [9]. SARS-CoV-2 is a Betacoronavirus closely related to other human pathogenic coronaviruses SARS-CoV and MERS-CoV that also emerged in the 21st century [10]. SARS-CoV-2 is an enveloped and non-segmented single-stranded positive-sense RNA virus having crown like spikes on the outer surface. The diameter and length of SARS-CoV-2 are about 65–125 nm and 29.9 kb respectively [11,12]. Structure of SARS-CoV-2 consists of 4 major proteins namely, spike (S) glycoprotein, envelope (E) glycoprotein, membrane (M) glycoprotein, and nucleocapsid (N) protein, as well as several non-structural and accessory proteins (see Figure 1). Among all the proteins, the spike (S) protein plays a key role in viral attachment, fusion, entry, and transmission. This S protein is responsible for the entry of SARS-CoV-2 into the host cell by attaching with angiotensin converting enzyme 2 (ACE2), which acts as a receptor and is present in different organs of the body [13,14].

COVID-19 is easily transmitted through saliva droplets or discharge from the nose of an infected person when he/she sneezes or coughs [15,16]. While most of the infected patients will show mild to moderate respiratory distress/illness and recover with or without requiring special treatments, the death toll number is shockingly high compared to other types of coronaviruses, SARS-CoV and MERS-CoV. The symptoms include fever, dry cough, shortness of breath, sore throat, tiredness, and aches and pains [2]. The Centers for Disease Control and Prevention (CDC) have identified some severe symptoms which require emergency medical attention including, but not limited to, persistent pain or pressure in the chest, trouble breathing, inability to wake or stay awake, new confusion, and bluish lips or face [17]. Although COVID-19 primarily affects the respiratory system, recent reports have revealed some neurological and cerebrovascular symptoms associated with the disease, including headache, disturbed consciousness, paresthesia [1], and, most recently, stroke [18]. Additionally, brain tissue edema and partial neuronal degeneration, as well as viral encephalitis attacking the central nervous system (CNS) have also been reported [19,20]. Therefore, it seems that COVID-19 can promote harm of the cerebrovascular system and the CNS of infected patients.

Smoking (cigarettes and cannabis) and vaping are significant public health concerns in the USA and around the globe. Ample studies have found that smoking is associated with different diseases affecting different organs of the body, many of which are fatal. Smoking is considered a risk factor for developing and progression of cancers and major forms of respiratory distress, including chronic obstructive pulmonary disease (COPD), pulmonary fibrosis etc. [21]. It has been reported that smoking is responsible for the development of squamous metaplasia in large airways, hypersecretion of mucus and hyperplasia in smooth muscle, along with increased small airway fibrosis and thickening of the airway wall which ultimately results in narrowing and destruction of the airway, accompanied by bronchitis. Patients may also suffer from airflow limitation due to emphysematous lung destruction [22]. Smoking is also considered a significant pro-immunogenic mix of substances impacting the immune responses and promoting the onset of autoimmune disorders (such as rheumatoid arthritis and systemic lupus) in genetically-susceptible individuals. Smoking severely impacts the vascular system promoting the onset of neurological diseases as well as fatal cardiovascular diseases [23]. By comparison with non-smokers, smokers are more prone to respiratory illness, including colds, increased rates of influenza, bacterial pneumonia, and tuberculosis [24]. Smoking causes damage to the lungs which, in turn, makes the patients more vulnerable to viral and bacterial pulmonary infections as well. Tobacco smoke causes structural changes, including peri-bronchiolar inflammation and fibrosis, enhanced mucosal permeability, impaired mucociliary clearance, changes in pathogen adherence, and disruption of the respiratory epithelium, which ultimately dysregulate immune defenses of respiratory system. Moreover, smoking is related to a wide range of alterations in cellular and humoral immune system function [25].

In comparison to non-smokers, smokers have an increased risk of developing influenza [25] and this infection can even exacerbate the comorbidities that are common in these populations [26]. It has also been reported that, after influenza infection, smokers are at higher risk of hospitalization compared to non-smokers [27]. Tobacco smoke not only plays a crucial role in developing respiratory distress but is also associated with an increased risk of cerebrovascular and neurological diseases, like stroke, Alzheimer’s disease, multiple sclerosis, and vascular dementia by disrupting the blood-brain barrier (BBB), inducing oxidative stress, inflammation, and the alteration of immune responses [28,29,30,31]. Noticeably, the negative effect of smoking on the progression of COVID-19 infection has been reported in recent case studies and reports [32,33,34,35]. As smoking increases the risk and susceptibility of SARS-CoV-2 infection, increasing the progression of COVID-19, and leading to severe respiratory distress and cardiovascular disease, this review article aims to determine plausible comorbid CNS and cerebrovascular roles of smoking and vaping in COVID-19 patients.

## 2. CoV Infection and CNS

Previous studies reported the disruption of the structure and function of the CNS due to viral infection resulting in severe encephalitis, toxic encephalopathy, and severe acute demyelinating lesions [1,36]. Some neurotropic viruses can cause infections of macrophages, microglia, and astrocytes by invading nervous tissues [37,38]. It is evident from previous studies that respiratory-related infections act as a critical factor for developing the acute cerebrovascular disease [39,40] Moreover, the influenza virus has been found to exacerbate ischemic brain injury through initiating cytokine cascade, thus increasing the probability of tissue-type plasminogen activator mediated cerebral hemorrhage [41].

SARS-CoV was found to cause different neurological diseases, including encephalitis, polyneuropathy, and large artery ischemic stroke [42]. Subsequently, the occurrence of cerebral edema and meningeal vasodilation along with the presence of the SARS-CoV genome sequence were identified in the brain of several SARS cases from autopsy studies [1,43]. Moreover, monocyte and lymphocyte penetration in the vessel wall, ischemic changes of neurons, and nerve fiber demyelination were also detected in autopsies of brain samples of the infected patients [43,44].

MERS-CoV, another coronavirus, causes Middle East Respiratory Syndrome or MERS and is known as neuroinvasive. It has been found from different studies that MERS-CoV is also responsible for causing different neurological complications, including insanity, seizures, ischemic stroke, paralysis, disturbed consciousness, Guillain-Barre syndrome, and other poisoning or infectious neuropathy [45,46].

## 3. Neurological and Cerebrovascular Manifestations of COVID-19

SARS-CoV-2, the responsible virus for COVID-19, is 79.5% genetically similar to SARS-CoV and 96% similar to bat coronavirus [47]. The sequence homology of SARS-CoV-2 also showed a 50% similarity to MERS-CoV virus [48]. Although the primary symptoms of COVID-19 include fever, dry cough, and fatigue in most of the patients [33], some COVID-19 patients exhibited sole neurological symptoms including headache, dizziness, languidness, unstable walking, malaise, cerebral hemorrhage, and infarction without showing any of the typical COVID-19 symptoms [49]. Additional studies have also reported a sudden loss of smell or taste in some COVID-19 patients as well [50,51].

A current study comprising 214 patients demonstrated that 36.45% of patients of the total cohort showed neurological symptoms, including acute cerebrovascular disease, impairment of consciousness, and skeletal muscle motor function disability; 18.7% of total admitted patients had these severe neurological manifestations and required admission to the intensive care unit [49,52]. Other case studies (shown in Table 1) also reported that acute cerebrovascular and neurological symptoms, including headache, dizziness, impaired consciousness, olfactory disorders, have been found in COVID-19 patients. Table 1 summarizes recent case studies related to COVID-19 and neurological dysfunction. However, one of the limitations of the case studies is that the analysis of cerebrospinal fluid (CSF) and electroencephalography (EEG) was not performed to confirm the presence of the virus in the CSF [53].

Another recent report has also shown COVID-19 to causes sudden stroke in patients aged between 30 and 40 years old. Although these COVID-19 infected patients had mild or no symptoms of COVID-19, abnormal blood clotting in large arteries has been reported, which ultimately resulted in severe stroke [18].

Another important finding is the detection of the genome sequence of SARS-CoV-2 in cerebrospinal fluid, which opens up a direction towards the damage of CNS in COVID-19 patients causing viral encephalitis [1]. Moreover, some of the COVID-19 patients were found to suffer from viremia and hypoxia [59], which play a crucial role in developing toxic encephalopathy. The occurrence of headache, disturbance in consciousness, other neurological dysfunction is close to 40% of COVID-19 patients [60], and the concurrent detection of brain tissue edema seems to suggest the existence of a possible link between COVID-19 and infectious, toxic encephalopathy [19]. However, extensive studies need to be conducted to validate this hypothesis further. Additionally, it has been reported that SARS-CoV-2 can initiate a cytokine storm mechanism, which may lead to a range of infectious and non-infectious diseases, including pancreatitis, acute cerebrovascular disease, and multiple organ dysfunction [61,62,63]. Critically-infected patients also showed a high level of D dimer and severe reduction in platelets, which may make the patients more vulnerable to acute cerebrovascular dysfunction [60,64]. Additionally, it has been speculated that COVID-19 positive patients are vulnerable to other types of pathogenic bacteria, which can damage the integrity of the blood-brain barrier (BBB). Subsequently, this secondary infection may lead to headaches, vomiting, loss of vision, and limb convulsions in COVID-19 patients [1].

Focusing on current case studies and research on COVID-19 patients, it is evident that COVID-19 could be associated with neurological and cerebrovascular dysfunction, which can be life-threatening as well.

## 4. Pathophysiology of COVID-19 Related Cerebrovascular and Neurological Dysfunction

Although the underlying mechanism behind cerebrovascular and neurological dysfunction in COVID-19 patients has not been elucidated yet, several potential mechanisms could be non-exclusively responsible for the identified comorbidities. One of the critical targets of SARS-CoV-2 is Angiotensin-converting enzyme 2 (ACE2) [49], which is present in different organs including lung, heart, kidney, testis as well neurons and glial cells of the brain [13,65,66,67]. ACE2 plays a pivotal role in the regulation of blood pressure as well as anti-atherosclerosis mechanisms [68]. It has been demonstrated from various studies that different types of CoV and influenza viruses may elevate blood pressure and increase the potential risk of cerebral hemorrhage by binding to ACE2. A recent study has also reported that SARS-CoV-2 enters into the host cell through the interaction of SARS-CoV-2 coat protein SPIKE or (S protein) with ACE2 present on the host cell resulting in the internalization of the virus [69,70,71]. The expression of ACE2 is found to be low in hypertensive patients, which increases the chance of hemorrhagic occurrence. Since SARS-CoV-2 decreases the ACE2 expression [72] it can be speculated that the SARS-CoV-2 infected patients are at high risk of hemorrhagic stroke (see Figure 1).

Additionally, COVID-19 patients suffer from coagulopathy and prothrombin time prolongation, which may contribute to secondary cerebral hemorrhage, although, as of today, no secondary cerebral hemorrhage has been reported in COVID-19 patients [49]. Moreover, an increased level of D-dimer has been found in COVID-19 patients, which may result in thrombotic vascular events [49,73]. As SARS-CoV-2 has been found in cerebrospinal fluid, it is crucial to evaluate the protective role of the BBB in preventing the virus from getting access to neural tissues [67]. This is of crucial importance since comorbid pathologies (such as those promoted by chronic smoking and vaping [28,31,74,75]) that negatively impact the integrity and function of the BBB may facilitate the virus entry into the CNS.

Another important mechanism behind the cerebrovascular and neurological symptoms in COVID-19 patients could be an immune injury. It has been found that viral infection may damage the nervous system by altering the immune responses [76]. A CoV infection-mediated severe pneumonia could promote systemic inflammatory response syndrome (SIRS). Studies suggested that immune damage could be prevented by early anti-inflammatory intervention and could also decrease the risk of nervous system injury [61,77]. Both SARS and COVID-19 have been found to cause multiple organ failure-mediated fatalities through virus-induced SIRS or SIRS-like immune disorders [62,78].

Cytokines play a pivotal role in regulating immunological and inflammatory function of the body [79]. Additional studies confirmed the release of high level of inflammatory factors, such as interleukin-6 (IL-6), interleukin-12 (IL-12), interleukin-15 (IL-15), and tumor necrosis factor-α (TNF-α) from primary glial cells infected with CoV [80]. Recently, Wan et al. reported the correlation of IL-6 with the severity of COVID-19 symptoms [81]. IL-6 may act as a potential biomarker of SARS-CoV-2 as IL-6 level has been found to be increased in COVID-19 patients [79] As CoV infection can infect macrophages, microglia, and astrocytes in the CNS inducing pro-inflammatory conditions [82] and activation of immune cells, it is crucial to find the probable correlation between COVID-19 and neurological damage through immune injury.

Moreover, the proliferation of viruses in the lung tissue may lead to an impaired exchange of alveolar gas, thus triggering hypoxia in CNS. This hypoxia causes anaerobic metabolism in brain cells, which accumulates acid causing cerebral vasodilation, brain cells swelling, interstitial edema, blockage of cerebral blood flow, and headache because of ischemia and congestion [83]. Untreated hypoxia may induce acute cerebrovascular disease encompassing acute ischemic stroke in high-risk COVID-19 patients [1]. As COVID-19 patients often suffer from fatal silent hypoxia, it requires substantial examination and consideration [84]. Additionally, ACE2 also plays a role in controlling inflammatory and atherosclerosis responses of vessels [85]. Thus, COVID-19 may promote atherosclerosis formation, which ultimately may result in brain ischemic stroke by affecting brain microcapillaries.

A neurotrophic virus can also enter the CNS through neuronal pathways such as the olfactory neuron transport system. Studies reported that, in the early stage of infection or nasal vaccination, CoV could reach the brain through the olfactory tract, thus causing inflammation and demyelination [1,86,87]. Therefore, it is evident that CoV viruses can invade the brain by neuronal pathways, and this mechanism should also be investigated in the case of SARS-CoV-2.

## 5. Smoking, COVID-19, and Cerebrovascular-Neurological Diseases

Tobacco smoking is responsible for a wide range of diseases affecting different organs of the body, including cerebrovascular, cardiovascular, pulmonary systems, and this can be life-threatening as well [88]. These diseases include, but are not limited to, lung cancer, chronic obstructive pulmonary disease (COPD), cardiovascular diseases, stroke, and decreased immune function [89]. Yearly, tobacco smoking (TS) kills around 6 million people in the world, and more than 0.48 million people in the USA alone [90]. Although the main addictive component of TS is nicotine, it also contains more than 4700 toxic compounds encompassing carcinogens, mutagens, stable and unstable free radicals as well as reactive oxygen species (ROS). Different studies demonstrated the association between tobacco smoke and cerebrovascular-neurological dysfunction, including ischemic stroke, Alzheimer’s diseases, multiple sclerosis, abnormal brain development, and vascular dementia [28,90,91,92]. The mechanisms behind the toxic effects of smoking include, but are not limited to, inflammation, oxidative stress, atherosclerosis, disruption of the BBB, and hyperactive immune response [29,30].

The BBB plays a pivotal role in maintaining brain homeostasis and acts as a strong shield to prevent the entrance of the potentially harmful substances from system blood circulating into the brain parenchyma. Moreover, it protects the brain by limiting the body’s peripheral immune defense system from entering inside the brain parenchyma [74]. Therefore, disruption of BBB integrity may expose the brain temporarily to potential hazardous components (both exogenous and endogenous) circulating in the blood, which may affect neuronal activities both in the CNS and the periphery [74,93]. Loss of BBB viability may, in turn, increase the risk for secondary brain damage and may progress the pathogenesis of a variety of CNS diseases such as epilepsy, silent cerebral infarction, hemorrhagic and non-hemorrhagic stroke, small vessel ischemic disease, and traumatic brain injury [74,94,95,96,97]. Different studies reported that TS causes endothelial dysfunction and damages the vascular system. TS acts as an inflammatory agent causing oxidative stress, which may be responsible for impairment of BBB [98,99,100,101]. Even at low concentrations, TS induces strong vascular pro-inflammatory responses. These encompass the upregulation of endothelial pro-inflammatory genes, pro-inflammatory cytokines such as Interleukin -1β (IL-1β), TNF-α, upregulation, and activation of matrix metalloproteinase-2 and -9 (MMP-2, MMP-9), and monocyte differentiation into macrophages [31,74]. MMP-2 and MMP-9 affect BBB integrity by degrading basal laminal components and facilitating immune cell trafficking into the brain [102]. All of these pathogenic events promote BBB dysfunction and breakdown, which increases the risk of cerebrovascular disease, including stroke and other neurological disorders [31,74]. Howkins et al. reported the downregulation of zonula occludentes-1 (ZO-1; a tight junction—TJ—accessory protein linking the TJs to the cellular actin cytoskeleton) at the BBB by nicotine, causing increased BBB permeability [103]. Additionally, other scientific studies demonstrated the a7nAChR mediated alteration of the function of BBB Na^+^ K^+^ 2Cl^-^ co-transporter by nicotine [104,105,106]. TS is also associated with the progression of atherosclerosis and angiogenesis [90]. Signal transducer and activator of transcription-3 (STAT-3) is an angiogenesis modulator that acts by IL-6/STAT-3 signaling mechanism and can be upregulated by TS [107]. TS may also upregulate Apo-lipoprotein E genes, which regulate the metabolism of lipoprotein and is related to increased cholesterol level [90]. This later can further elevate the risk of ischemic stroke and atherosclerosis. Serum Amyloid A1 (*SAA1*) gene expression can also be upregulated by TS, which may subsequently increase the BBB permeability [108,109].

Furthermore, tobacco smoke also generates a high amount of superoxide, hydrogen peroxide, hydroxyl radical, and peroxynitrite, which exposes endothelial cells to highly reactive oxygen species (ROS), leading to oxidative stress (OS) damage [110]. OS ultimately results in lipoperoxidation of polyunsaturated fatty acids in membrane lipids, protein oxidation (backbone fragmentation), DNA breakdown [111,112,113], mutations of the nuclear protein p53, carcinogen-mediated DNA damage, RNA oxidation, mitochondrial depolarization, dysregulation of iron transporters and detoxifying enzymes, and apoptosis [28,114]. These pathological effects may further impair the cerebrovascular integrity and function, along with other factors or infectious agents that affect BBB [28,74,75,100,101].

Smokers are more vulnerable to bacterial and viral inflammatory neuropathologies compared to non-smokers [88] and have been shown to promote cerebral vasodilation along with reduced BBB integrity. Therefore, it is not surprising that chronic smokers are more susceptible to CNS disorders and, overall, neuronal damage caused by infection [115,116]. It has also been shown that the post-deep brain stimulation neuronal infection rate is higher in smoking patients compared to the non-smoking patients [117]. These observed detrimental effects in smokers could be caused by several mechanisms like increased inflammation and ROS, which leads to leaky BBB, and increased expression of receptors that promote virus invasion into the brain parenchyma. Recently, Brake et al. reported that smoking can upregulate the ACE2 receptor [24], which acts as a binding site for the S protein of SARS-CoV, coronavirus NL63, and SARS-CoV-2. This is the first immunohistochemical human lung evidence for ACE2 receptor expression in smokers and patients with COPD which identified the increased level of ACE2 expression in resected lung tissue from patients with COPD and healthy lung function smokers while entirely absent in healthy non-smoking individuals. As COPD patients showed significantly higher levels of ACE2, suggesting that COPD further exaggerates ACE2 and potential SARS-CoV-2 adhesion site [24]. Another recent study demonstrated the dose-dependent upregulation of ACE2 in a subset of epithelial cells lining the respiratory tract which includes goblet cells, club cells, and alveolar type 2 cells by cigarette smoke. This study also suggested that, smokers are more likely to develop SARS-CoV-2 infection compared to non-smokers [118]. Moreover, a recent in vitro study has shown that SARS-CoV-2 can infect engineered human blood vessels organoids, and this interaction can be inhibited by human recombinant soluble ACE2 (hrsACE2) antibody, thus highlighting a possible venue to treat COVID-19 [119].

**Figure 2:**
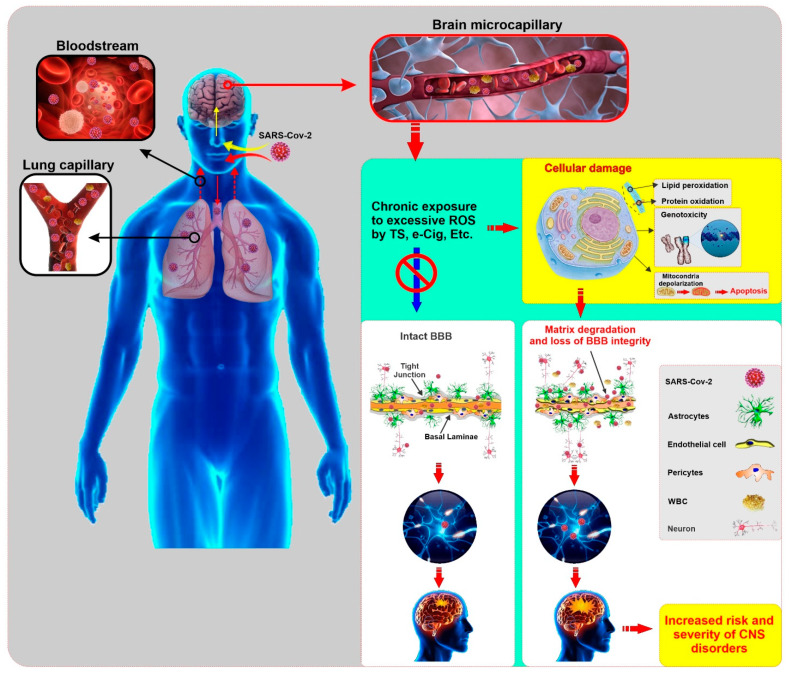
Illustrative panel summarizing the SARS-CoV-2 entry into the human body and the potential impact of comorbid smoking and/or vaping on the harmful effects of the viral infection to the CNS. Pre-existing conditions that impairs the viability and function of the BBB (such as those associated with chronic smoking and/or vaping) may facilitate viral entry into the brain, thus increasing the risk of onset and severity of CNS disorders. (ROS: Reactive Oxygen Species, TS: Tobacco Smoke, e-cig: electronic cigarette, BBB: Blood Brain Barrier, WBC: White Blood Cell, CNS: Central Nervous System)

In addition to lung, kidney, heart, and intestine, this receptor is also expressed in endothelial cells, glial cells, and neurons, which could increase the risk and progression of COVID-19 [120,121]. Thus the cerebral involvement of COVID-19 can result from the dissemination of the virus into the systemic circulation from the infected organ, which has been reported in other SARS-CoV affected patients [122]. Impaired BBB due to chronic smoking may facilitate the entry of the SARS-CoV-2 virus into brain parenchyma. Later, this invading virus can interact with neuronal and glial ACE2 receptors and start a viral proliferation cycle, which ultimately causes neuronal damage as previously observed in SARS-CoV [122]. Therefore, it can be reasonably speculated that smoking may enhance the risk for COVID-19 by upregulating the ACE2 [24] and promoting the loss of BBB integrity and viability (see Figure 2).

In addition to ACE2, another crucial pro-coagulant factor, von Willebrand factor (VWF), is upregulated in COVID-19 patients [123,124]. On top of that, previous epidemiological studies have shown that smoking increases the circulatory level of VWF. VWF is a glycoprotein and exclusively synthesized by endothelial cells and megakaryocytes. Functionally, VWF is responsible for carrying factor VIII in blood circulation and also mediates initial platelet adhesion to the subendothelium through glycoprotein Ib-IX complex after inflammation and injury [125]. In addition to VWF upregulation, smoking has been recently shown to promote the downregulation of thrombomodulin [75,126]. Thrombomodulin acts as an anticoagulant factor by binding to thrombin and use its enzymatic activities to degrade factor V, thus blocking the prothrombinase complex. The ultimate effect is a significant alteration of the blood homeostasis with a significant propensity toward blood coagulation and an increase in the risk of ischemic disorders like stroke [127,128]. Additionally, microthrombi were found in the circulation of several organs in COVID-19 patients, which has been claimed to be generated due to the dissemination of intravascular coagulation (DIC) [129,130,131]. Thus, it is highly likely that these microthrombi can also reside inside the microvascular system of the CNS and ultimately results in neurological complications. As smoking can increase the level of VWF and decrease the level of thrombomodulin, there could be a correlation between smoking and stroke occurrence in COVID-19 patients. However, extensive well-designed and controlled animal experiments are required to confirm this hypothesis.

Additionally, a recent study has suggested that smoking may promote cellular uptake of SARS CoV-2 virus through α7nAChR signaling mechanism. As α7nAChR is present both in neuronal and non-neuronal cells, therefore it can be said that, smoking may play a vital role in pathophysiology of SARS-CoV-2 and may affect different organs of the body including brain [132].

## 6. Vaping (E-Cigarette), COVID-19, and Cerebrovascular-Neurological Diseases

At the present time, electronic cigarettes, or e-cigarettes, have become extremely popular among youth on the pretense to be a safe alternative to tobacco smoke. It delivers nicotine by heating a vape liquid containing nicotine, flavoring agents, and different solvents into an aerosol [133]. The aerosol contains different harmful components including (but not limited) flavoring agents, humectants (such as glycerin and propylene glycol), contaminants (such as heavy metals), and harmful solvent byproducts (including formaldehyde and acrolein) in addition to tobacco specific nitrosamines [133,134]. All these substances can harm the cerebrovascular systems and the BBB in a way not too dissimilar from TS [133,134]. In fact, Kaisar et al. recently reported that chronic e-cigarette smoking is responsible for disrupting the BBB integrity and promote vascular inflammation. Moreover, it may facilitate the onset of stroke and worsen the condition of post-ischemic brain injury [75]. Another recent study also reported that e-cigarette vaping may decrease neuronal glucose utilization, which could result in increased risk for ischemic brain injury and stroke [135]. Recently, McAlinden et al. suggested that, nicotine-based e-cigarettes or vaping may contribute to the upregulation of ACE2 which may also play an important role in progression and outcome of COVID-19 [136].

## 7. Cannabis, COVID-19, and Cerebrovascular-Neurological Diseases

Cannabis, or marijuana, is the most widely abused recreational drug around the world, which is associated with cerebrovascular and neurological diseases such as stroke, structural and functional changes in the brain, cognitive and behavioral disorders [137]. Compared to TS and e-cigarette, smoking cannabis can also generate ROS and Δ-9-tetrahydrocannabinol (THC), the main component of cannabis which promotes OS as well as inflammation that may result in the onset of ischemic stroke [138,139]. Other possible mechanisms behind cerebrovascular-neurological dysfunction related to cannabis smoking include among others cerebral vasoconstriction, cerebral artery luminal stenosis, cerebral auto-dysregulation, and angiopathy [140,141,142,143]. A recent report demonstrated that smoking cannabis could deteriorate the condition of COVID-19 patients through airway inflammation [144]. Although no case has been reported on cerebrovascular dysfunction in COVID-19 patients and smoking cannabis yet, cannabis could be a risk factor for developing neurological disorders in COVID-19 patients due to its detrimental effect on the cerebrovascular system.

## 8. Conclusions

The COVID-19 pandemic has taken a tremendous hit on the individuals and family lives all around the world within a short period. This has left health care providers and researchers unprepared and with a plethora of questions to be answered. For instance, who are more vulnerable to this infection? What are the risk factors associated with the severity of this infection? How to tackle the severity and widespread of this kind of infection in the future?

Most importantly, what are the organs that could get affected by SARS-CoV-2 infection as we need to take care of the current as well as recovered patients in the future. From the above-mentioned case reports, it can be speculated that this virus can affect the CNS along with the lung, heart, and gastrointestinal system. These case reports show the presence of neurological disorders in as high as 36.4% COVID-19 patients [52]. Yet, the mechanism of CNS invasion by COVID-19 is unknown. One of the probable hypotheses is that it can reach the brain through the olfactory nerve system present in the nasal cavity [145]. However, the presence of SARS-COV, a family member of *Coronavirus*, in the CSF suggested an alternative mechanism of CNS invasion for this class of viruses [146]. Since the BBB protects the brain parenchyma from viral and bacterial infection, damage to this biological barrier could also lead to the accumulation of deadly viruses like SARS-CoV-2 in the CNS. Several studies, including in our lab, have shown the detrimental effect of tobacco and e-cigarette smoking on BBB integrity. Thus, it can be speculated that smoking could lead to the increased severity of SARS-CoV-2 infection by affecting the viability and integrity of the BBB while promoting the expression levels of ACE2 (the responsible mediator of SARS-CoV-2 cell invasion and proliferation) in endothelial cells, glia, and neurons. Furthermore, increased blood circulatory level of VWF and decrease levels of thrombomodulin promoted by smoking and vaping can dysregulate the blood homeostasis promoting blood coagulation and the formation of unwanted blood clot which severely increases the risk of stroke and cardiovascular disorders. At this stage, it is clear that additional studies will be necessary to validate these hypotheses, including further analyses of autopsy samples from smoking and non-smoking COVID-19 patients or conducting in vivo studies. K18-hACE2 transgenic mouse developed by McCray et al. for SARS-CoV studies along with tobacco smoke exposure rodent models could be useful available animal models for studying pathogenesis of SARS-CoV-2 and evaluating the impact of smoking and vaping on cerebrovascular and neurological dysfunction in COVID-19 patients [147,148].

## Figures and Tables

**Table 1:** Case studies on neurological and cerebrovascular symptoms in COVID-19 patients.

Study Type	Time	Study Design	Outcome and Symptoms	Reference
Retrospective case series	13 January to 31 March	*N* = 274, admitted patients	Headache (11.31%), Dizziness (7.66%)	[54]
Retrospective case series	16 January 2020 to 29 February 2020	*N* = 221, admitted patients	Acute ischemic stroke (5%), CVST (0.5%), cerebral hemorrhage (0.5%)	[55]
Retrospective case series	16 January 2020 to 19 February 2020	*N* = 214, admitted patients	Nervous system symptoms (36.4%) including CNS symptoms (24.8%): (Headache (13.1%), dizziness (16.8%), impaired consciousness (7.5%), acute cerebrovascular disease (2.8%), ataxia (0.5%), epilepsy (0.5%))	[52]
Retrospective case series	1 January to 28 January, 2020	*N* = 138 admitted patients	Headache (7%), dizziness (9%)	[56]
Retrospective case series	1 January to 20 January, 2020	*N* = 99, admitted patients	Headache (8%), confusion (9%)	[57]
Cross-sectional survey	19 March, 2020	*N* = 59, admitted patients	Headache (3.4%) Taste or olfactory disorder (33.9%), Taste and olfactory disorder (18.6%)	[50]
Retrospective case series	late December 2019- 26 Jan 2020	*N* = 52, admitted patients (critically ill adults)	Headache (6%)	[58]
Prospective case series	By 2 January 2020	*N* = 41, admitted patients	Headache (8%) in 38 patients	[33]
Case study	23 March to 7 April	*N* = 5	Large-vessel stroke (100%)	[18]

## Abbreviations

ACE2	Angiotensin-converting enzyme 2
BBB	Blood–brain barrier
BMVEC	Brain microvascular endothelial cells
CDC	Centers for Disease Control and Prevention
CNS	Central nervous system
COPD	Chronic Obstructive Pulmonary Disease
DIC	Dissemination of intravascular coagulation
e-cig	Electronic cigarette
IL-1β	Interleukin -1β
IL-6	Interleukin-6
IL-12	Interleukin-12
IL-15	Interleukin-15
MERS	Middle East Respiratory Syndrome
MMP-2	Matrix metalloproteinase-2
MMP-9	Matrix metalloproteinase-9
OS	Oxidative stress
ROS	Reactive oxygen species
SARS	Severe acute respiratory syndrome-related coronavirus
SIRS	Systemic inflammatory response syndrome
STAT-3	Signal Transducer and Activator of Transcription-3
TBI	Traumatic brain injury
THC	Δ-9-tetrahydrocannabinol
TNF-α	Tumor necrosis factor-α
TJ	Tight junction
TS	Tobacco smoking
VWF	Von Willebrand factor
WBC	White blood cell
WHO	World Health Organization
ZO-1	Zonulae occludentes-1
